# From online engagement to offline action: how social media environmental engagement shapes university students’ pro-environmental citizenship through intrinsic motivation and personal norms

**DOI:** 10.3389/fpsyg.2026.1868354

**Published:** 2026-06-19

**Authors:** Yuchen Gao, Qi Li, Yu Han, Jian Xu

**Affiliations:** 1 School of Business Administration, Anhui University of Finance and Economics, Bengbu, China; 2 School of Statistics and Applied Mathematics, Anhui University of Finance and Economics, Bengbu, China

**Keywords:** environmental intrinsic motivation, environmental self-accountability, organizational citizenship behavior for the environment, self-determination theory, social media environmental engagement, value-belief-norm theory

## Abstract

**Introduction:**

In the context of intensifying global ecological crises, leveraging social media platforms to foster spontaneous pro-environmental behaviors among college students has become a critical issue in sustainability research. This study integrates self-determination theory and the value-belief-norm theory to construct a serial dual-mediator model, with environmental intrinsic motivation and environmental self-accountability as sequential mediators. It examines how social media environmental engagement influences university students’ organizational citizenship behavior for the environment and the underlying psychological mechanisms.

**Methods:**

Based on survey data from a sample of Chinese university students, this study employs regression analysis and the bootstrap method for mediation testing.

**Results:**

The results support three coexisting mediation pathways: the separate mediating role of environmental intrinsic motivation, the separate mediating role of environmental self-accountability, and the serial mediating role of environmental intrinsic motivation followed by environmental self-accountability.

**Discussion:**

These findings reveal a deep alignment between the motivational internalization mechanism of self-determination theory and the norm-activation mechanism of the value-belief-norm theory. Specifically, the deep internalization of intrinsic motivation systematically amplifies the effect of norm activation. Practically, these insights provide a basis for designing digital strategies for environmental education in universities and crafting content for environmental communication on social media.

## 1 Introduction

Social media are profoundly reshaping the relationship between young people and environmental issues. According to data from the China Internet Network Information Center, the average daily time that Chinese university students spend on social media exceeds 3 h. Within this time, browsing, sharing, and discussing environmental content have become routine online activities. According to recent findings, social media exposure to environmental issues strongly influences how students feel about the environment and the actions they intend to take (Tong et al., 2024). Among Generation Z, this tendency is especially noticeable, given their generally strong level of participation (Confetto et al., 2023). However, a notable gap persists. Despite young people’s growing online environmental participation, their spontaneous offline pro-environmental behaviors have not increased to a comparable degree. The effect is most noticeable in organizational citizenship behavior for the environment (OCBE)—that is, voluntary environmental efforts that extend beyond formal job duties (Boiral and Paillé, 2012). This phenomenon can be viewed as a form of environmental “slacktivism” (Kristofferson et al., 2014) and reflects the broader attitude-behavior gap in environmental psychology (Kollmuss and Agyeman, 2002). The disconnect between online enthusiasm and offline action presents both a practical challenge and a theoretical puzzle. Recent research has confirmed that social media marketing by environmental organizations can effectively stimulate pro-environmental behavior (Bilgin and Çetinkaya, 2025), yet the psychological mechanisms underlying this process remain underexplored. This raises a key question: through what psychological pathways does social media environmental engagement (ESME) translate university students’ online behaviors into voluntary pro-environmental actions in real-world settings?

Although existing research provides preliminary evidence for two theoretical pathways, prior studies have already identified multiple mechanisms (e.g., environmental knowledge, awareness, and responsibility), but these mechanisms are typically examined in isolation rather than within an integrated framework, three theoretical extensions have not been simultaneously examined to the above question. On one hand, self-determination theory (SDT) (Deci and Ryan, 2013) suggests that external stimuli can promote the internalization of environmental motivation by satisfying needs for competence, autonomy, and relatedness (Klein, 2019). Meanwhile, the value-belief-norm theory (VBN) (Stern et al., 1999) shows a clear chain: awareness of consequences and ascription of responsibility work together to activate personal norms, which then guide behavior (Steg and Vlek, 2009). However, three gaps remain. First, although limited research has begun to uncover the psychological pathways through which taking part in social media can translate into pro-environmental behavior outside the digital space (Han M. S. et al., 2025), most existing studies focus on single mediators such as environmental knowledge or responsibility, and the underlying sequential mechanisms have not yet been systematically integrated in a single model. Few studies have systematically unpacked the psychological pathways that lead to OCBE in organizational settings. Second, the motivational internalization mechanism from SDT and the norm-activation mechanism from VBN have consistently been tested as independent pathways (van Riper et al., 2020). Whether these two mechanisms form a sequential chain—that is, whether the formation of intrinsic motivation provides a cognitive prerequisite for activating responsibility norms—remains lacking direct empirical evidence. Consequently, existing models have missed the incremental effects arising from the synergy between the two mechanisms. Third, OCBE research has long focused on workplace contexts (Daily et al., 2009). Unlike Han Z. et al. (2025), who focused on organizational climate and green self-efficacy in campus settings, our study examines a digital antecedent (ESME) and introduces a dual-pathway mechanism based on self-determination theory (for environmental intrinsic motivation, EIM) and value-belief-norm theory (for environmental self-accountability, ESA). We focus on how motivational and normative mechanisms interact under high voluntariness and low external constraints in social media contexts, a question that has not been addressed in prior OCBE research on Chinese university students, rather than merely extending existing models to a new setting.

The psychological pathways through which social media participation drives OCBE among university students have not yet been empirically tested in this specific integration (Zhang and Huang, 2025). These three gaps collectively point to the core question of this study: between ESME and OCBE among university students, how do EIM and ESA sequentially mediate the relationship, and does their chain synergy produce incremental effects beyond each single pathway?

To address the research questions above, this study builds a serial dual-mediator model that uses EIM and ESA as two mediators in sequence. This model captures a long-overlooked theoretical connection between SDT and VBN. When university students frequently engage with and share environmental content on social media, they gradually gain a sense of competence and autonomy. As a result, their pro-environmental behavior shifts from external compliance to internalized value identification, forming genuine EIM (Klein, 2019). However, motivational internalization is not the end of the psychological process. Rather, it serves as a critical cognitive foundation for activating the VBN norm-based pathway. Only when environmental values are sufficiently internalized and stable do individuals become more acutely aware of the consequences of environmental problems. They then attribute environmental responsibility to themselves, develop stable ESA, and ultimately engage in spontaneous pro-environmental behaviors that exceed formal requirements (Stern et al., 1999). Accordingly, this study proposes that university students’ ESME promotes their OCBE through three parallel psychological pathways. The first is the single pathway via EIM alone (the SDT pathway). The second is the single pathway via ESA alone (the VBN pathway). The third is the sequential pathway through “EIM—ESA” (the integrated pathway combining SDT and VBN). This chain path differs from earlier integrated studies (such as Duong et al., 2024). Duong et al. integrated self-determination theory, norm activation model and planned behavior theory in the context of sustainable consumption, but their model did not set a clear theoretical sequence between motivation internalization and individual norm activation. In contrast, we proposed a theory-driven sequence, where EIM precedes ESA, and EIM provides the basis for the activation of ESA. Furthermore, our outcome variable is OCBE, which is a voluntary behavior beyond roles and conceptually differs from consumption behavior.

The theoretical contributions of this study lie in three main areas. First, it clarifies the psychological mechanisms through which ESME influences university students’ OCBE. Within a single analytical framework, this study simultaneously tests two independent pathways—motivational internalization and norm activation—as well as their sequential synergy. In doing so, it provides a full psychological pathway account of how young people’s pro-environmental behaviors develop in the digital media era. Second, it provides empirical support for the cross-theoretical integration of SDT and VBN. The research has revealed the chain mechanism that “internalization of intrinsic motivation is the cognitive prerequisite for normative activation,” breaking the fragmented testing paradigm of the two theories in previous studies and supplementing the detailed explanation of the front-end motivation conditions for the single normative activation path of the VBN theory. Third, expand the research scenarios of OCBE to the group of Chinese college students, focusing on ESME, a new type of influencing factor with the characteristics of The Times. This not only accumulates new empirical evidence for the cross-context applicability of this construct, but also provides practical references based on psychological mechanisms for environmental protection education in colleges and universities and the intervention design of social media platforms.

## 2 Theoretical basis and research hypotheses

### 2.1 Model development

**Figure 1:**
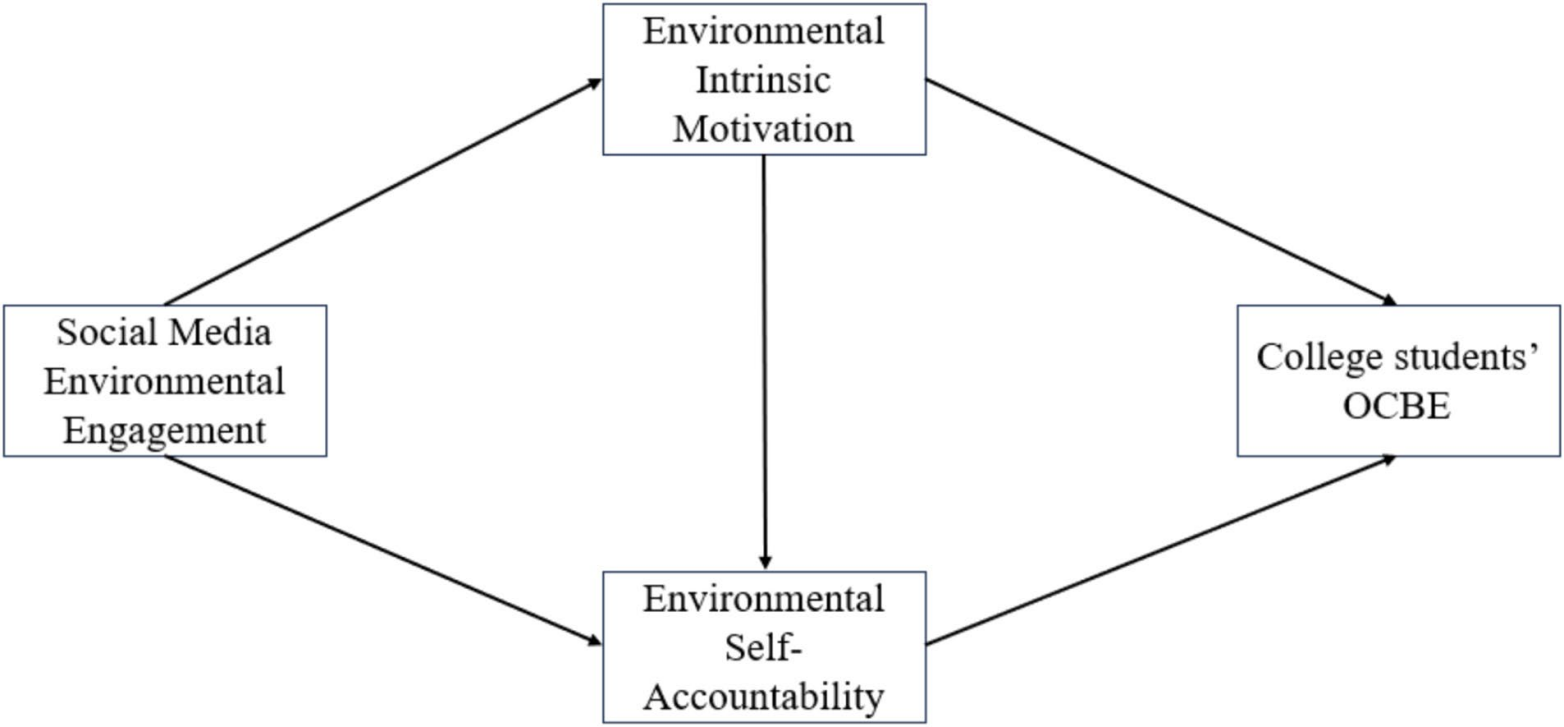
Theoretical model.

This study integrated SDT (Deci and Ryan, 2013) and VBN (Stern et al., 1999) to construct a chain double-mediating model of ESME influencing OCBE in college students (see Figure 1). SDT points out that when the external environment meets an individual’s three basic psychological needs of competence, autonomy and social connection, external regulation will be transformed into intrinsic motivation through the internalization process, driving continuous autonomous behavior (Ryan and Deci, 2000; Gagné and Deci, 2005). Unlike external incentives, intrinsic motivation-driven behaviors do not rely on rewards and punishments and have stronger spontaneity and persistence (Gagné and Deci, 2005; Boiral et al., 2015), which makes it a key psychological mechanism for explaining superrole voluntary behaviors such as OCBE. VBN reveals the normative activation path of pro-environmental behavior: After individuals are exposed to environmental protection information, through the progressive activation of consequence awareness and responsibility attribution, a sense of moral obligation for action (personal norms) is formed, and it is transformed into spontaneous pro-environmental actions (Steg and Vlek, 2009). This study operationalizes the fusion construct of responsibility attribution and personal norms as “ESA” to capture the psychological process by which college students internalize environmental responsibility as self-moral restraint. When individuals form a true intrinsic value recognition of environmental protection behaviors, their perception of environmental consequences becomes more acute, responsibility attribution becomes more stable, and the activation of ESA also has a deeper cognitive basis (Stern et al., 1999; Gagné and Deci, 2005). Conceptually, EIM reflects value internalization (“I truly care”), whereas ESA reflects moral obligation (“I have a duty to act”). Theoretically, EIM serves as a cognitive foundation for activating ESA, justifying the serial mediation model over a parallel-only model. Based on this, this study predicts three coexisting mediating paths, which constitute the theoretical model of this research.

### 2.2 Research hypotheses

#### 2.2.1 The mediating role of EIM

ESME refers to the total sum of behaviors of individuals actively browsing, commenting on, forwarding and creating environmental-related content on social media platforms (Boulianne and Ohme, 2022). According to SDT, this participation behavior can meet the basic psychological needs of college students through three parallel paths, thereby promoting the internalization of environmental protection motives. First, the path of competence. Through continuous exposure and sharing of environmental protection issues, college students gradually accumulate environmental protection knowledge and action skills, and form the efficacy cognition of “I have the ability to understand and participate in solving environmental problems” (Xin et al., 2026). Second, the path of social connection. The environmental participation behaviors of peer groups on social media have a normative demonstration effect, and college students form a group identity of “I belong to this environmental community” in peer interactions (Pelletier et al., 1998). Third, the path of sense of autonomy. Unlike the one-way indoctrination of traditional media, social media grants users the right to independently choose content, express opinions and set the depth of participation, enabling college students to participate in environmental protection issues in a self-determined way (Klein, 2019).

Together, these three pathways address students’ basic psychological needs. As a result, their psychological regulation of pro-environmental behavior shifts from “others expect me to participate” to a genuinely internalized value orientation—"I truly care about the environment and voluntarily want to take action” (Toussard and Meyer, 2025). The university years represent a crucial phase for value development and identity formation among students. They are particularly sensitive to social information about the environment, and their rate of internalizing external regulation is higher than that of other age groups (Zsóka et al., 2013). Once pro-environmental behavior becomes an integral part of their self-identity, students voluntarily go beyond formal role expectations. They then engage in OCBE on campus in spontaneous ways (Boiral and Paillé, 2012). In light of the reasoning presented above, this study puts forth:

*H1*: EIM significantly mediates the relationship between ESME and university students' OCBE.

#### 2.2.2 The mediating role of ESA

The VBN theory predicts that the occurrence of pro-environmental behavior can be achieved not only through the motivational internalization path but also through an independent normative activation path (Cao et al., 2022). Social media, with its characteristics of high frequency, strong emotion and wide coverage information dissemination, provides an ideal situation for the activation of this normative path (Ju et al., 2022). College students’ continuous exposure to environmental issues on social media, such as clips from climate change documentaries, visualized pollution incidents, and environmental advocacy content from their peers, will significantly enhance their awareness of the environmental consequences caused by human activities. Unlike the abstract data presentation of traditional media, the emotional narrative nature of social media content and the high credibility of peer sources make the activation of consequence awareness more direct and profound (Vraga and Bode, 2020). The strengthening of consequence awareness further triggers responsibility attribution: When college students realize that there is a causal relationship between their own behavior and environmental damage, the moral cognition of “I have the responsibility to take action” emerges and gradually solidifies into stable personal norms, forming ESA.

The core feature of ESA lies in that the mechanism driving its behavior stems from internalized moral self-standards rather than external social pressure or expectations from others. College students with a high degree of ESA will view their participation in OCBE as the fulfillment of their own moral commitment rather than compliance with external norms (Steg and Vlek, 2009). This behavioral driving force based on moral self-restraint has a unique predictive power for superrole voluntary civic behavior (Boiral and Paillé, 2012). It should be pointed out that the above-mentioned normative activation path is theoretically independent of the SDT’s motivational internalization path: even if an individual has not completed the full internalization of intrinsic motivation, a clear understanding of consequences and responsibilities alone is sufficient to activate self-responsibility and drive behavior (Steg and Vlek, 2009). This reasoning leads us to propose:

*H2*: ESA significantly mediates the relationship between ESME and university students' OCBE.

#### 2.2.3 The serial mediating role of EIM and ESA

This study further proposes that there is a sequential causal relationship between EIM and ESA. In the ESME, an individual gradually acts on the OCBE through this continuous path. According to the organic integration theory in SDT, when intrinsic motivation is highly internalized, individuals will truly integrate environmental protection behaviors into their own value systems, thereby forming a profound value recognition (Klein, 2019). And this deep recognition of value also lays the foundation for activating the VBN specification path from two aspects. Firstly, in terms of cognitive depth, the research by Aviste and Niemiec (2023) shows that intrinsic motivation is positively correlated with ecocentric values, and ecocentric values can enhance individuals’ perception of the consequences of environmental issues, providing a more comprehensive cognitive basis for responsibility judgment. This also means that individuals with stronger intrinsic motivation will conduct more systematic and in-depth thinking and processing when confronted with environmental-related information. Secondly, in terms of value stability, Tedaldi et al. (2024) conducted a cross-national study involving 31 countries and found that an individual’s perceived sense of responsibility has a stable predictive effect on their climate-related cognition and attitudes. Even after controlling for demographic variables, this relationship remains significant. It can be seen from this that the stability of responsibility attribution is closely related to the degree of internalization of value: the higher an individual’s internalization of environmental protection value, the more inclined they are to take the initiative to assume environmental responsibility rather than attribute it to external factors, and the level of ESA will also increase accordingly.

If college students’ participation in environmental protection only remains at a superficial level and is influenced by the outside world (such as forwarding environmental protection content under the pressure of peer atmosphere, rather than from the bottom of their hearts), then their understanding of environmental responsibility is very likely to waver, and it is difficult for them to truly form a stable and conscious sense of environmental responsibility (Zhu and Chen, 2024). It can be seen from this that the chain mediating effect proposed by H3 is not a simple superposition of the H1 and H2 paths, but rather reflects an incremental effect where “the deeper the internalization, the stronger the normative activation effect.” The degree of internalization of an individual’s intrinsic motivation will affect the intensity and sustainability of the normative activation path, making this chain path exhibit a unique indirect effect distinct from two independent mediating paths. This leads us to propose:

*H3*: EIM and ESA play a significant chain mediating role between ESME and OCBE of college students. That is, ESME is successively conducted through the sequence of EIM and ESA, exerting an indirect influence on OCBE of college students.

## 3 Research method

### 3.1 Sample and data collection

Data were collected online through Credamo using a three-wave longitudinal design. This platform features stringent data quality control mechanisms and has been widely used in behavioral and social research. Data collection spanned 5 weeks, from January 15 to February 15, 2026, with a 2-week interval between successive waves. The 2-week interval was chosen to reduce recall bias and common method variance while remaining short enough to minimize attrition and capture the theorized psychological processes of motivational internalization and norm activation. This temporal separation helped reduce common method bias by distancing the measurement of predictors from that of criterion variables. The first survey (T1) was launched on January 15, 2026, measuring demographic variables (gender, grade level, academic major, and daily social media use duration) and the independent variable (ESME). Questionnaires were distributed through the Credamo sample panel to university students enrolled in multiple institutions across China. This wave brought in 584 responses. Following preliminary screening, all 584 responses remained valid and served as the initial tracking sample. Two weeks later (T2), the second survey was sent to this sample, measuring the mediators (EIM and ESA). This wave yielded 515 valid responses. After another 2 weeks (T3), the third survey assessed the dependent variable (OCBE among university students), returning 420 valid responses. Participants were tracked across waves using unique user IDs assigned by the Credamo platform, and responses from T1, T2, and T3 were matched based on these IDs. Invalid responses were excluded based on the following criteria: (a) excessively short completion time for any single wave (<180 s); (b) failure to pass any embedded attention check item; (c) logical contradictions in responses to reverse-scored items; and (d) highly uniform response patterns (e.g., selecting the same option consecutively) or systematic zigzag patterns. After all the matching and screening, 374 participants made up the final valid sample. Attrition analysis showed that of the 584 participants at T1, 374 completed all three waves (retention rate = 64.0%). No significant differences were found between retained and dropped participants in gender, grade, or T1 ESME scores (all *p* > 0.05), indicating no systematic attrition bias.

In this sample, 22.2% were male and 77.8% female. Grade distribution was as follows: 6.1% first-year, 17.6% second-year, 21.7% third-year, 29.1% fourth-year, and 25.5% master’s students or above. Environment-related majors accounted for 5.1%, while non-environmental majors comprised 94.9%. Participants who used social media for more than 3 h per day made up 66.6% of the sample. The sample covered multiple universities in eastern, central, and western China, including comprehensive, science and engineering, and normal universities. We used a convenience sampling approach. Overall, the sample primarily consisted of female students, those from non-environmental majors, and heavy social media users. This composition provided a suitable data basis for examining the effects of ESME in this study.

### 3.2 Variable measurement

Every variable was rated on a 5-point Likert scale, from 1 (strongly disagree) to 5 (strongly agree). To ensure semantic equivalence between the Chinese and English versions, scales originally developed in English were translated using Brislin's (1980) two-way translation procedure. Three experts in environmental psychology assessed content validity and confirmed item relevance.

ESME: His study adapted the Adolescent Social Media Involvement Scale (Ni et al., 2020) and the Facebook Intensity Scale (Ellison et al., 2007) to the context of environmental topics. The original scales looked at the degree to which individuals incorporate social media into their daily routines. In this study, they were adapted to assess the degree to which university students engage with environmental content on social media. The adapted scale consists of four items covering behaviors such as browsing, sharing opinions, discussing topics, and liking or commenting. The specific items are: “I often browse and read environment-related content on social media”; “I actively share environmental information or opinions on social media”; “I often discuss environmental topics with others on social media”; and “I regularly like, comment on, or share environment-related content on social media.” The Cronbach’s *α* for this scale was 0.841 in the current study.

EIM: The EIM Scale in this study came from Han Z. et al. (2025). The original scale captures the inner motivation to act pro-environmentally, coming from genuine interest and shared values. This study directly adopted the three items that have been validated among Chinese university students. These items capture dimensions such as sense of meaning, enjoyment, and self-improvement. The specific items are: “I engage in pro-environmental behavior because it is very meaningful”; “I engage in pro-environmental behavior because it brings me joy”; and “I engage in pro-environmental behavior because it makes me a better person.” The Cronbach’s *α* coefficient for this scale came out at 0.730 in the current study.

ESA: The Self-Accountability Scale from Peloza et al. (2013) was modified for environmental issues in this study. The original scale measures perceived self-responsibility in consumption contexts. We adapted it to the environmental protection context for use with university students. The adapted scale consists of five items, one of which is reverse-scored. It covers dimensions such as perceived responsibility, behavioral intention, and moral obligation. The specific items are: “I feel I have a great deal of responsibility for environmental protection”; “I have a strong desire to assume social responsibility and care for the environment”; “Environmental sustainability does not have much to do with me” (reverse-scored); “I strongly want to contribute to environmental protection through my own actions”; and “I have an obligation to care for the natural environment.” The Cronbach’s *α* coefficient for this scale was 0.775 in the current study.

OCBE: This study used the OCBE Scale developed by Boiral and Paillé (2012). Following Han Z. et al. (2025) in their study of Chinese university students, the measurement context was adapted from the workplace to the campus setting. The original scale comprises three dimensions: eco-helping, eco-initiatives, and eco-civic engagement. The adapted version consists of six items measuring university students’ voluntary pro-environmental behaviors on campus. Example items include: “I take an active part in environmental activities and projects put on by my school or class “; “I voluntarily take environmental measures in my daily life (e.g., sorting trash, saving water and electricity)”; and “Even when it is not my responsibility, I voluntarily remind and advise fellow students to engage in environmental protection.” This scale produced a Cronbach’s *α* of 0.789 in our study.

Control variables: Following prior studies (Carducci et al., 2021), this study controlled for demographic characteristics that may influence university students’ pro-environmental behavior. These included gender (1 = male, 2 = female), grade level (1 = first year, 2 = second year, 3 = third year, 4 = fourth year, 5 = master’s student or above), academic major (1 = environment-related, 2 = non-environment-related), and daily social media use duration (1 = less than 1 h, 2 = 1–3 h, 3 = 3–5 h, 4 = more than 5 h).

### 3.3 Analytical strategy

Confirmatory factor analysis (CFA) was first performed using AMOS 29.0 in this study. By comparing different models, we examined the discriminant validity among the four core variables: ESME, EIM, ESA, and OCBE. We also evaluated the measurement model for goodness-of-fit. Next, descriptive statistical analysis was performed using SPSS software. Means, standard deviations, reliability coefficients (Cronbach’s *α*), and intercorrelations among variables were calculated. These findings laid the groundwork for testing our hypotheses. Finally, the hypotheses were examined using Model 6 of the PROCESS macro (Version 3.3) within SPSS. The analysis centered on testing the mediating roles played by EIM and ESA.

## 4 Results

### 4.1 Confirmatory factor analysis

We used AMOS 29.0 to run a CFA and check discriminant validity across the four main variables. As shown in Table 1, four alternative models were compared. The four-factor model fit the data better than any other model (*χ*^2^ = 258.648, df = 129, *χ*^2^/df = 2.005, CFI = 0.950, TLI = 0.941, RMSEA = 0.052, SRMR = 0.046). All fit indices reached acceptable thresholds, indicating that common method bias was not a significant concern. As presented in Table 2, the Cronbach’s α coefficients for the variables ranged from 0.730 to 0.841, and composite reliability (CR) values ranged from 0.729 to 0.848. Every score came in higher than the 0.70 benchmark, which means the internal consistency reliability was good. For convergent validity, ESME’s average variance extracted (AVE) came out at 0.584—above the 0.50 benchmark. Although the AVE values for EIM, ESA, and OCBE were slightly below this standard, their CR values were all above 0.70. According to Fornell and Larcker (1981), convergent validity can still be considered acceptable under these conditions. Discriminant validity was assessed using the heterotrait-monotrait (HTMT) ratio of correlations. All HTMT values among the constructs were within the range of 0.590 to 0.836, below the recommended threshold of 0.85, thereby demonstrating good discriminant validity. Additionally, the Fornell-Larcker criterion was satisfied: the square root of AVE for each construct exceeded its inter-construct correlations. In summary, the measurement model demonstrated satisfactory reliability and validity, supporting subsequent hypothesis testing.

**Table 1:** Comparison of measurement models.

Model	*χ* ^2^	df	*χ*^2^/df	CFI	TLI	RMSEA	SRMR
Four-factor model (ESME, EIM, ESA, OCBE)	258.648	129	2.005	0.950	0.941	0.052	0.046
Three-factor model (ESME+EIM, ESA, OCBE)	439.638	132	3.331	0.882	0.863	0.079	0.067
Two-factor model (ESME+EIM + ESA, OCBE)	557.031	134	4.157	0.837	0.814	0.092	0.067
Single-factor model (ESME+EIM + ESA + OCBE)	598.303	135	4.432	0.822	0.798	0.096	0.068

*N* = 374; ESME, Social Media Environmental Engagement; EIM, Environmental Intrinsic Motivation; ESA, Environmental Self-Accountability; OCBE, Organizational citizenship behavior for the environment.

**Table 2:** Reliability and validity.

Variables	*α*	CR	AVE	HTMT-EIM	HTMT-ESA	HTMT-OCBE
1. ESME	0.841	0.848	0.584	0.623	0.590	0.734
2. EIM	0.730	0.729	0.473		0.791	0.799
3. ESA	0.775	0.778	0.421			0.836
4. OCBE	0.789	0.800	0.400			

*N* = 374.

### 4.2 Common method Bias

Common method bias was assessed using Harman’s single-factor test. An exploratory factor analysis of the main study variables showed that, with eigenvalues greater than one and without rotation, the first principal component explained 39.409% of the variance. This value is below the 40% threshold, indicating that common method bias did not significantly affect the results. A confirmatory factor analysis model (M1) was then constructed, followed by a model that included a common method factor (M2). Comparing the key fit indices between M1 and M2 yielded the following changes: ∆*χ*^2^/df = 0.42, ∆CFI = 0.025, ∆TLI = 0.025, and ∆RMSEA = 0.012. The changes in CFI and TLI were both below 0.10, and the change in RMSEA did not exceed 0.05. These results suggest that adding a common method factor did not substantially improve model fit. Thus, no significant common method bias was present in the measurements. Finally, CFA was used to conduct common method bias tests for all self-assessed items. All the questions were classified into a common factor to construct a single-factor model. The results showed that the model fitted very poorly (*χ*^2^/df = 4.432, CFI = 0.822, TLI = 0.798, RMSEA = 0.096), and all the fitting indicators failed to meet the acceptable standards, and were significantly worse than the four-factor measurement model. This indicates that the variation of all variables cannot be explained by a common factor, further proving that there is no serious common method bias in this study.

### 4.3 Correlation analysis

Table 3 shows that ESME had strong positive correlations with EIM (*r* = 0.491, *p* < 0.01), ESA (*r* = 0.509, *p* < 0.01), and OCBE (*r* = 0.604, *p* < 0.01). EIM was also positively linked to ESA (*r* = 0.552, *p* < 0.01) and OCBE (*r* = 0.602, *p* < 0.01). Likewise, ESA correlated positively with OCBE (*r* = 0.620, *p* < 0.01). These findings gave us an initial green light for testing our hypotheses.

**Table 3:** Means, standard deviations, and correlations among variables (*N* = 374).

Variables	*M*	SD	1	2	3	4	5	6	7
1. Gender	1.780	0.416	1						
2. Grade	3.480	1.211	−0.006	1					
3. Major	1.950	0.220	0.023	−0.029	1				
4. Time	2.950	0.797	0.134^**^	−0.101	0.030	1			
5. ESME	3.089	0.714	−0.077	−0.076	−0.155^**^	0.014	1		
6. OCBE	3.731	0.520	−0.005	−0.006	−0.050	0.007	0.604^**^	1	
7. EIM	4.011	0.516	−0.030	−0.018	0.005	0.002	0.491^**^	0.602^**^	1
8. ESA	3.535	0.357	0.009	−0.011	0.013	0.005	0.509^**^	0.620^**^	0.552^**^

^*^p < 0.05, ^**^p < 0.01.

### 4.4 Hypothesis testing

This study used regression analysis and PROCESS macro Model 6 (Hayes, 2017) to test the serial mediation model. The model examined how ESME influences university students’ OCBE through EIM and ESA. Bootstrap resampling was performed 5,000 times. The results are presented below.

First, the regression results (see Table 4) showed that after controlling for gender, grade level, academic major, and daily social media use duration, ESME significantly and positively predicted EIM (*β* = 0.507, t = 11.013, *p* < 0.01) and also significantly and positively predicted ESA (*β* = 0.332, *t* = 6.909, *p* < 0.01). Further results showed that EIM had a significant positive effect on ESA (*β* = 0.390, *t* = 8.261, *p* < 0.01). Using OCBE as the dependent variable in the regression model, ESME (*β* = 0.314, *t* = 7.144, *p* < 0.01), EIM (*β* = 0.280, *t* = 6.343, *p* < 0.01), and ESA (*β* = 0.306, *t* = 6.823, *p* < 0.01) all had significant positive effects on OCBE. These results indicated that all path coefficients among the variables were significant, providing the necessary basis for testing mediation effects.

**Table 4:** Test of the serial mediation model of EIM and ESA.

Model	OCBE		EIM		ESA		OCBE	
	*β*	*t*	*β*	*t*	*β*	*t*	*β*	*t*
Gender	0.043	1.024	0.009	0.191	0.045	1.094	0.026	0.716
Grade	0.043	1.024	0.023	0.503	0.022	0.536	0.027	0.755
Major	0.046	1.099	0.084	1.840	0.062	1.490	−0.007	−0.183
Time	−0.005	−0.107	−0.007	−0.156	−0.006	−0.149	0.001	0.007
ESME	0.618	14.658^***^	0.507	11.013^***^	0.332	6.909^***^	0.314	7.144^***^
EIM					0.390	8.261^***^	0.280	6.343^***^
ESA							0.306	6.823^***^
*R* ^2^	0.370		0.249		0.385		0.548	
*F*	43.279^***^		24.379^***^		38.332^***^		63.320^***^	

^***^p < 0.01.

Second, the Bootstrap mediation results based on PROCESS Model 6 (see Table 5) showed that all indirect pathways had 95% confidence intervals that excluded zero. Specifically, the indirect effect of ESME on OCBE via EIM was 0.100, with a 95% confidence interval of [0.062, 0.143]. Accounting for 22.73% of the total effect, this indirect effect indicated that EIM served as a significant mediator. Accordingly, Hypothesis H1 received support. The indirect effect of ESME on OCBE through ESA was 0.070 (95% CI [0.041, 0.104]). This accounted for 15.91% of the total effect, supporting Hypothesis H2. Furthermore, the serial indirect effect via the pathway ESME—EIM—ESA—OCBE was 0.044, with a 95% confidence interval of [0.025, 0.067]. Since zero wasn’t in the interval, and the effect accounted for 10.00% of the total effect, it was meaningful. These results suggest that EIM and ESA played a significant sequential mediating role between ESME and OCBE, supporting Hypothesis H3.

**Table 5:** Mediating effect values and effect sizes in the serial mediation model.

Effect type	Mediation path	Effect value	95% CI	Effect size
Mediating effect	X → M1 → Y	0.100	[0.062, 0.143]	22.73%
Mediating effect	X → M2 → Y	0.070	[0.041, 0.104]	15.91%
Mediating effect	X → M1 → M2 → Y	0.044	[0.025, 0.067]	10.00%
Total indirect effect		0.214	[0.161, 0.270]	48.64%
Total effect		0.440	[0.381, 0.499]	100.00%

Effect size, Effect value of each path/total effect.

**Figure 2:**
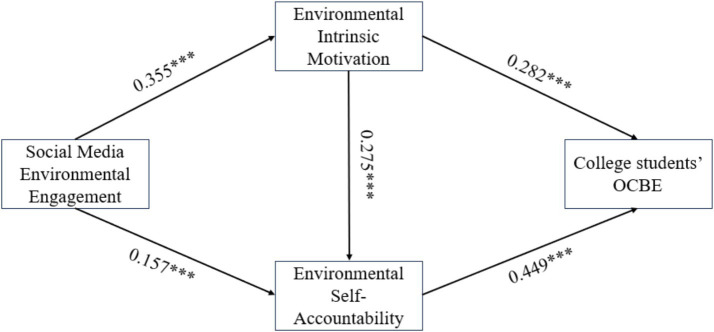
Path diagram of the serial mediation structural equation model.

Regarding overall effects, the total effect of ESME on OCBE was 0.440. With a value of 0.214, the total indirect effect explained 48.64% of the total effect. This suggested that mediation played an important role in the relationship. The direct effect remained significant, indicating that the model represents partial rather than full mediation. As illustrated in Figure 2, all path coefficients were positive and significant. These results showed that ESME not only directly promoted OCBE among university students but also indirectly fostered OCBE by enhancing EIM, which in turn strengthened ESA. In summary, all three research hypotheses received empirical support, and the serial mediation model demonstrated good fit.

## 5 Discussion and conclusion

Drawing on SDT and VBN as theoretical frameworks, this study validated three mediating pathways through which ESME drives OCBE among Chinese university students. Hypotheses H1, H2, and H3 were all supported by the data. These findings indicate that the effect of ESME on students’ OCBE operates through the independent and sequential mechanisms of motivational internalization and norm activation. Regarding H1, ESME satisfies students’ needs for competence, autonomy, and relatedness. This facilitates the internalization of external regulation into EIM, which in turn stimulates their willingness to go beyond role boundaries and voluntarily engage in OCBE. This finding aligns with Klein's (2019) core proposition that external environments satisfying basic psychological needs promote the internalization of extrinsic regulation into autonomous regulation, thereby motivating extra-role behavior. The significance of this pathway among university students echoes Wullenkord's (2023) argument that basic psychological needs in educational contexts foster autonomous behavior. At the same time, it extends this mechanism to the digital context of pro-environmental behavior, broadening the applicability of SDT in informal digital settings (Frank and Kohn, 2023).

The high emotional narrativity of social media, combined with the high credibility of peer sources, also strengthens students’ awareness of environmental consequences. Through stable responsibility attribution, this process generates an internalized sense of moral obligation that independently drives their extra-role voluntary actions. This finding supports and extends the VBN framework proposed by Stern et al. (1999). Compared with the abstract data narratives typical of traditional media, the emotional nature of social media content and the trustworthiness of peer sources make the activation of consequence awareness and responsibility attribution more direct and profound (Liao, 2025; Ajina et al., 2024). This reveals a unique reinforcing effect of the social media context on the VBN norm-activation pathway. Schwartz's (1977) norm-activation theory has long indicated that consequence awareness and responsibility attribution are key antecedents to activating personal norms and forming moral obligations. This study further validates and deepens this mechanism in the social media context. Taken together, these findings suggest that the unique attributes of social media contexts can more effectively activate the VBN norm-activation chain, thereby promoting university students’ voluntary pro-environmental behaviors that go beyond role boundaries.

The serial indirect effect was found to be significant and distinct from the two separate mediation pathways. This indicates that deep internalization strengthening norm activation constitutes a distinct explanatory mechanism with incremental predictive power. Nevertheless, this serial pathway should be interpreted as complementary rather than dominant, given its modest effect size relative to the two independent pathways. As an individual’s level of internalized EIM increases, the more acute their perception of environmental consequences, the more stable their responsibility attribution, and the stronger the activation of ESA. This finding breaks with prior research paradigms that have examined SDT and VBN separately. It provides empirical support for the integrative mechanism linking the two theories. Moreover, it suggests that examining only single mediation pathways would fail to fully capture the psychological process through which ESME drives OCBE, and would likely overlook the incremental explanatory power of theoretical integration.

### 5.1 Theoretical contributions

First, this study helps bring SDT and VBN together in a meaningful way. Unlike prior integrations such as Duong et al. (2024) which did not impose a specific theoretical order between the two mechanisms, our model tests a theoretically mandated sequence: intrinsic motivation precedes personal norm activation. Further analysis showed that the serial indirect effect in this study was statistically independent of the two single mediation pathways (Liu and Tang, 2025; Šakan et al., 2020). Thus, the explanatory power of the integrated pathway arises from the synergy between the two theories rather than from a simple summation of their separate effects. We acknowledge that this serial mechanism plays a complementary rather than dominant role alongside the two independent pathways. Cao et al. (2022), when integrating VBN with the theory of planned behavior, also noted that intrinsic motivation is a key factor influencing behavioral intentions. This conclusion indirectly supports the present approach—placing the SDT motivational pathway before the VBN normative pathway. In summary, this study provides empirical support for developing a more complete integrated theory of pro-environmental behavior (Carfora et al., 2021). It also offers a refinement to VBN theory: personal norm activation is not a moral reaction that emerges in a vacuum but is cognitively predicated on the degree of internalized intrinsic motivation.

Second, this study expands the psychological antecedents and research context of OCBE. Existing research has largely focused on organizational-level antecedents such as leadership behavior and organizational climate (Han M. S. et al., 2025; Wu et al., 2025), primarily using workplace employees as participants (Hussain et al., 2025). This has neglected the antecedent activation mechanisms among university students in educational settings. Indeed, Han Z. et al. (2025) explicitly noted that while OCBE has been extensively examined in organizational contexts, empirical research targeting university students remains insufficient. This study extends OCBE research to Chinese university students, identifying EIM and ESA as two core internal psychological mechanisms driving this behavior. This approach is consistent with Hou et al. (2026), who used a student sample and validated the significant predictive effects of intrinsic green motivation and perceived environmental responsibility on green behavioral intentions. Similarly, Aviste and Niemiec (2023), drawing on SDT, found that intrinsic life aspirations positively influence pro-environmental behavioral intentions. These findings fill a research gap concerning university students and offer a new perspective on the nomological network of antecedents of OCBE, suggesting that its psychological driving mechanisms are stable across contexts.

Third, this study reveals a dual-pathway mechanism through which ESME influences pro-environmental behavior. Existing research has largely remained at the level of direct “exposure—behavior” effects, with insufficient attention to underlying psychological mediation mechanisms (Chen, 2020). Moreover, existing mediation studies have mostly been confined to single theoretical frameworks (Cao et al., 2022; Gkargkavouzi et al., 2019). This study simultaneously tested two mediation pathways—SDT and VBN—and their serial effect within a single model. It establishes motivational internalization and norm activation as two core psychological mediators linking ESME to offline pro-environmental actions. This approach echoes Hou et al. (2025), who distinguished two pathways in social media digital green intentions: external recognition orientation versus internal satisfaction orientation. It is also theoretically isomorphic with Liang et al. (2026), who identified imitation intention as a core mechanism translating social media influencer effects into pro-environmental behavior. These findings provide a new theoretical interface for interdisciplinary research on digital communication and pro-environmental behavior, deepening the mechanistic understanding of social media engagement research.

### 5.2 Practical implications

First, universities should rethink the design philosophy of environmental education participation. This study found that merely increasing exposure to environmental information—such as through lectures or distributing promotional materials—has limited effects on activating students’ OCBE. These methods do not meet students’ psychological needs for competence, autonomy, and relatedness. Universities should instead create environmental education scenarios that are interactive, participatory, and community-oriented. Examples include establishing student-led environmental social media accounts, building campus-based pro-environmental practice communities, and organizing online environmental content creation competitions. These strategies can effectively activate the internalization process of intrinsic motivation. At the same time, contextualized responsibility narratives can strengthen students’ ESA, thereby achieving synergistic activation of both pathways.

Second, the psychological targeting of environmental communication content should be optimized. The validation of the serial pathway suggests that environmental communication should address two types of psychological objectives. One is to provide knowledge-based and competence-oriented information that satisfies recipients’ need for competence and facilitates motivational internalization. The other is to adopt concrete and attributable responsibility framing—for example, “how my daily behaviors affect the environment”—to activate recipients’ awareness of consequences and ascription of responsibility, thereby reinforcing personal norms. Communication content that simultaneously activates both types of mechanisms can produce synergistic behavioral effects through the serial pathway that go beyond either single pathway alone. Accordingly, environmental communication organizations could design a sequential communication strategy that presents cognitive construction first, followed by responsibility narratives, to enhance the effectiveness of content in activating students’ OCBE.

Third, digital environmental participation should be incorporated into university sustainability governance systems. This study demonstrates that ESME has significant indirect effects on students’ OCBE through three pathways. This finding indicates that digital participation channels are important institutional resources for activating students’ spontaneous pro-environmental actions. Universities could integrate ESME into extracurricular practice credit systems, establish campus-based environmental content creation communities, and set up online discussion platforms for environmental issues. These measures can provide systematic institutional support for the synergy between motivational internalization and norm activation, thereby more effectively translating online participation into sustained voluntary pro-environmental actions.

### 5.3 Limitations and future directions

First, although this study employed a three-wave longitudinal design, causal inference remains limited. The temporal separation of measurements (T1: ESME, T2: EIM and ESA, T3: OCBE) provides stronger evidence for the hypothesized causal sequence than a cross-sectional design would. However, we cannot completely rule out the possibility of reverse causality. For example, students with higher levels of OCBE may be more inclined to engage with environmental content on social media. Future research could adopt experimental designs to conduct more rigorous causal tests.

Second, there is the potential influence of common method bias. The use of a single self-report questionnaire to measure all variables introduces a potential risk of common method bias. Although procedural controls such as anonymous responses, spaced presentation of scales, and statistical tests were implemented, future research could further reduce this bias by using multiple data sources. Examples include peer-rated measures of OCBE or objective behavioral records.

Third, there is the limitation of external validity due to a single cultural context. Our sample consisted of Chinese university students, so it’s still unclear whether the findings hold across different cultures. China’s collectivist cultural background may have strengthened the facilitating effect of relatedness on intrinsic motivation internalization. The effect size of the motivational internalization pathway may therefore differ in individualist cultural contexts. Moreover, the sample was predominantly female (77.8%) and non-environmental majors (94.9%), which may further limit generalizability. Future research could adopt multi-country comparative designs to systematically test the moderating role of cultural value orientations (collectivism vs. individualism) on the path coefficients in this model.

Fourth, boundary conditions and moderating effects remain to be explored. This study examined only mediation effects and did not investigate boundary conditions that may moderate the three pathways. For example, type of social media use (active participation vs. passive browsing) may moderate the effect of ESME on EIM. Individuals’ level of environmental concern may moderate the effect of ESA on OCBE. Students’ academic major and grade level may influence the relative effect sizes of the three pathways. Future research could introduce moderated mediation models to systematically reveal the heterogeneity and boundary conditions of the three pathway effects, thereby further refining the theoretical model proposed in this study.

Fifth, the set of control variables is limited. We only controlled for gender, grade, major, and daily social media use. Key covariates from prior research were not included, such as environmental knowledge, environmental concern, social desirability, campus green climate, prior participation experience, and family socioeconomic status. Future research should incorporate these variables to rule out potential unmeasured confounding.

## 6 Conclusion

Grounded in an integrated framework of SDT and VBN, this study empirically tested a sample of Chinese university students. It systematically revealed how ESME drives students’ OCBE through the dual pathways of EIM and ESA. The results demonstrated that the motivational internalization pathway from SDT and the norm-activation pathway from VBN each have independent mediating effects. Moreover, the serial synergistic effect whereby deep internalization amplifies norm activation further uncovers the deeper integration mechanism between the motivational and normative psychological systems in driving voluntary pro-environmental actions. The simultaneous support for all three pathways suggests that adopting only a single-pathway model would systematically underestimate the complete psychological process through which social media engagement drives students’ OCBE. This discovery not only provides a new integrated theoretical framework for understanding the psychological mechanisms of pro-environmental behaviors among young people in the digital age, but also offers precise strategic references based on psychological mechanisms for the practical design of environmental education in colleges and universities and environmental protection communication on social media.

## Data Availability

The raw data supporting the conclusions of this article will be made available by the authors, without undue reservation.
